# Both living bacteria and eukaryotes in the mosquito gut promote growth of larvae

**DOI:** 10.1371/journal.pntd.0006638

**Published:** 2018-07-06

**Authors:** Luca Valzania, Vincent G. Martinson, Ruby E. Harrison, Bret M. Boyd, Kerri L. Coon, Mark R. Brown, Michael R. Strand

**Affiliations:** Department of Entomology, The University of Georgia, Athens, GA, United States of America; University of Wisconsin Madison, UNITED STATES

## Abstract

We recently reported that larval stage *Aedes aegypti* and several other species of mosquitoes grow when living bacteria are present in the gut but do not grow when living bacteria are absent. We further reported that living bacteria induce a hypoxia signal in the gut, which activates hypoxia-induced transcription factors and other processes larvae require for growth. In this study we assessed whether other types of organisms induce mosquito larvae to grow and asked if the density of non-living microbes or diet larvae are fed obviate the requirement for living organisms prior results indicated are required for growth. Using culture conditions identical to our own prior studies, we determined that inoculation density of living *Escherichia coli* positively affected growth rates of *Ae*. *aegypti* larvae, whereas non-living *E*. *coli* had no effect on growth across the same range of inoculation densities. A living yeast, alga, and insect cell line induced axenic *Ae*. *aegypti* first instars to grow, and stimulated similar levels of midgut hypoxia, HIF-α stabilization, and neutral lipid accumulation in the fat body as *E*. *coli*. However, the same organisms had no effect on larval growth if heat-killed. In addition, no axenic larvae molted when fed two other diets, when fed diets supplemented with heat-killed microbes or lysed and heat-killed microbes. Experiments conducted with *An*. *gambiae* yielded similar findings. Taken together, our results indicate that organisms from different prokaryotic and eukaryotic groups induce mosquito larvae to grow, whereas no conditions were identified that stimulated larvae to grow in the absence of living organisms.

## Author summary

Studies conducted in our laboratory indicate that several species of mosquito larvae require living bacteria in their gut to grow. Previous results also show that living bacteria reduce gut oxygen levels below 5% and that bacteria-induced gut hypoxia functions as a signal that activates several processes in larvae with growth functions. Here we report that bacterial density affects larval growth rates and that other living organisms besides bacteria induce larval stage *Aedes aegypti* and *Anopheles gambiae* to grow. In contrast, the requirement for living organisms was not altered by the diet larvae were fed. These findings are of fundamental interest because they show that several types of organisms in the gut activate the same growth-related processes when viable but none do so when non-viable. Reported results also have applied implications for producing axenic adult mosquitoes for use in different types of studies.

## Introduction

Mosquitoes are aquatic during their juvenile stages and grow by feeding on organic detritus, unicellular organisms and small invertebrates [1,2]. Larval and adult stage mosquitoes also host a community of living microbes in their digestive tract that form a gut microbiota [3,4]. Laboratory studies indicate that mosquito larvae hatch with no gut microbes but rapidly acquire a microbiota through persistence of a subset of the microbes they consume [5,6]. Most identified community members are gram-negative aerobic or facultatively anaerobic bacteria [5–13] although several unicellular eukaryotes including fungi and algae are also known [14–16]. Results from different studies further indicate that species diversity of the gut microbiota is relatively low in both larval and adult stage mosquitoes but exhibits high intra- and interspecific variation as a function of collection locality and other factors [3–18].

*Aedes aegypti* is a broadly distributed container-breeding species with adult females being important vectors of several human pathogens [1]. *Ae*. *aegypti* larvae reared under conventional (non-sterile) conditions molt through four instars before pupating and harbor a microbiota of ~100 bacterial species in our own laboratory culture [1,19]. We previously reported that axenic *Ae*. *aegypti* larvae with no gut microbiota consume a rat chow-based diet we normally use for rearing, but do not grow and after many days die as first instars [5]. *Escherichia coli* and several other species of bacteria that have been identified as gut community members in different populations of *Ae*. *aegypti* [5,16] individually colonize the guts of axenic larvae, which results in gnotobiotic larvae that develop like conventionally reared individuals [5]. However, axenic larvae were not rescued if fed rat chow diet and inoculated with dead bacteria or cultured in water containing rat chow diet that had been preconditioned by living bacteria [5]. F1 progeny from field-collected *Ae*. *aegypti* plus several other mosquito species exhibited the same defects under axenic conditions, while development was similarly rescued if larvae were inoculated with *E*. *coli* or other gut community members [5,18]. Studies from other laboratories have reported similar outcomes [20]. We thus collectively interpreted these results as evidence that several mosquito species require living microbes in their gut to develop.

Within each instar, *Ae*. *aegypti* larvae grow by feeding until they achieve a critical size, which induces feeding to cease and the titer of the steroid hormone 20-hydroxyecdysone to rise which stimulates molting [19,21]. Bioassays showed that conventional larvae or gnotobiotic larvae inoculated with wild-type *E*. *coli* exhibit reduced gut oxygen levels (hypoxia) when precritical size and are feeding but exhibit normoxia when postcritical size and have stopped feeding [21]. Results further showed that bacteria primarily pass through the gut when larvae feed with little or no loss of viability but exhibit greatly reduced viability in postcritical size larvae due to protracted exposure to high midgut pH [21]. In contrast, axenic larvae or larvae inoculated with bacteria defective for aerobic respiration feed but exhibit gut normoxia in conjunction with not achieving critical size or molting [21]. These results strongly suggested that aerobic respiration by bacteria in the gut reduce oxygen levels in precritical size larvae but retention and pH-mediated mortality of bacteria in postcritical size larvae cause oxygen levels to rise. They further indicated that most ingested microbes do not establish residency but instead are iteratively acquired and lost between instars. We thus hypothesized that living aerobic or facultatively anaerobic bacteria generate a phasic gut hypoxia response that functions as a growth signal for larvae in each instar [21]. We further reasoned that transduction of such a signal could involve dimeric (α/ß) hypoxia-induced transcription factors (HIFs), which modulate responses to oxygen through processes that stabilize HIF-α under hypoxia but degrade HIF-α under normoxia [22–24]. Consistent with these suggestions, we detected stabilized HIF-α in *Ae*. *aegypti* larvae that exhibited bacteria-induced gut hypoxia and grew but did not detect HIF-α in axenic larvae that exhibited gut normoxia and did not grow [25]. Functional experiments further implicated HIF signaling in regulation of several processes with functions in nutrient acquisition and metabolism [25]. In contrast, larvae do not molt normally when exposed to environmental hypoxia [21], which suggests a requirement for localized microbe-induced hypoxia in the gut for normal growth.

Since mosquito larvae ingest other unicellular organisms besides bacteria, we previously suggested some could induce larvae to grow by activating a gut hypoxia signal [21]. However, mosquito larvae can also be reared on other diets besides what we used in past studies, which could alter the requirement for living microbes our previous experiments indicated. For example, axenic *Anopheles stephensi* larvae were reported to grow on a diet comprised primarily of liver extract and brewer’s yeast (*Saccharomyces cerevisiae*) [26] while axenic *An*. *stephensi* and *Toxorhynchites amboinensis* larvae grew when fed an established insect cell line (RU TAE 12 V) in Leibovitz (L-15) medium [27]. These findings suggest that bacteria are not specifically required for growth of mosquito larvae but leave open the possibility aerobic respiration in the gut by other types of organisms could promote larval growth like bacteria. Alternatively, the diets used in these studies could provide nutrients or other factors that make living organisms unnecessary for growth. In this vein, axenic *Ae*. *aegypti* larvae were recently reported to grow into adults when fed a diet comprised of liver powder and yeast extract if supplemented with high densities of heat-killed *E*. *coli* or *S*. *cerevisiae*, which suggest the combination of diet and dead microorganisms could overcome a requirement for living organisms in the gut [28].

We therefore examined in this study three factors that could affect the role of gut microbes in growth of *Ae*. *aegypti* and other mosquitoes. First, we characterized the growth of *E*. *coli* under culture conditions used in our previous studies and assessed whether the density of living versus non-living bacteria in a culture affects larval growth. Second, we compared *E*. *coli* to other aerobic organisms that larvae can ingest to ask whether they can promote growth. Third, we tested whether other diets used to rear mosquitoes allow *Ae*. *aegypti* and *Anopheles gambiae* larvae to grow in the absence of living organisms in the midgut. Our results indicated that several other organisms besides bacteria induced gut hypoxia, HIF-α stabilization, and larval growth. However, no axenic larvae grew when inoculated with non-living organisms over a range of densities and diets.

## Materials and methods

### Ethics statement

Animal care and use are described in Animal Use Protocol A2018 02-002-Y1-AO (renewal 2/27/2018), which was approved by The University of Georgia Institutional Animal Care and Use Committee (IACUC). The UGA IACUC oversees and provides veterinary care for all campus animal care facilities and is licensed by the US Department of Agriculture (USDA) and maintains an animal welfare Assurance, in compliance with Public Health Service policy, through the NIH Office of Laboratory Animal Welfare, and registration with the USDA APHIS Animal Care, in compliance with the USDA Animal Welfare Act and Regulations, 9 CFR. IACUC personnel attend to all rodent husbandry under strict guidelines to insure careful and consistent handling. The University of Georgia’s animal use policies and operating procedures facilitated compliance with applicable federal regulations, guidance, and state laws governing animal use in research and teaching including the: 1) The Animal Welfare Act, 2) Public Health Service (PHS) Policy on the Humane Care and Use of Laboratory Animals, 3) United States Government Principles for the Utilization and Care of Vertebrate Animals Used in Testing, Research and Training, 4) Guide for the Care and Use of Laboratory Animals, 5) Guide for the Care and Use of Agricultural Animals in Research and Teaching, 6) American Veterinary Medical Association Guidelines for the Euthanasia of Animals, and 7) Applicable Georgia laws.

### Microbes and S2 cell line

Wild-type *E*. *coli* (K12 strain) were grown in Luria broth (LB) (Difco) at 37° C, wild-type *S*. *cerevisiae* were grown at 30° C in YPD broth (Thermo Fisher), and wild-type green alga *Chlamydomonas reinhardtii* (g1) cells were grown at 21° C in minimal (M) medium with a 14 h light:10 h dark photoperiod [29]. The S2 cell line from *Drosophila melanogaster* was maintained in 10 cm^2^ culture flasks (Corning) and passaged weekly in serum-free SFX medium (HyClone) [30]. Growth of *E*. *coli* or *S*. *cerevisiae* was monitored by measuring optical density (OD) at 600 nM followed by determination of cell density using a Neubauer hemocytometer to generate growth curves for each. An OD of 1.0 equaled ~1 x 10^9^ cells per ml for *E*. *coli* and ~6 x 10^7^ cells per ml for *S*. *cerevisiae*. *C*. *reinhardtii* and S2 cell density in a given culture were determined directly using a hemocytometer. All living cell types were in the log phase of growth when used in bioassays. Living bacteria and yeast were pelleted at 2000 x *g* and resuspended in sterile water to a specific density before use in bioassays (see below). Whole *E*. *coli* or *S*. *cerevisiae* cells resuspended in water were either heat killed directly by autoclaving or lysed on ice by sonication using a Fisher model 100 sonicator (40% amplitude using four 5 sec pulses) before autoclaving. Autoclaved cells and lysates were then either used directly or immediately aliquoted and stored at -80° C before use. Assays using *E*. *coli* or *S*. *cerevisiae* were conducted in water. Assays using *C*. *reinhardtii* were conducted in M medium while assays using S2 cells were conducted in SFX medium. *C*. *reinhardtii* and S2 cells were heat killed in M or SFX medium respectively by incubation for 1 h at 70° C.

### Mosquito diets

Three main diets were used in bioassays: 1) a 1:1:1 mixture (wt/wt) of laboratory rat chow (LabDiet 5001), lactalbumin (Fisher) and torula yeast (US Biochemicals), 2) TetraColor tropical fish food granules (Tetra), and 3) 3 g of liver powder (MP Biomedical) plus 2 g of dried yeast extract (Fisher) in 100 ml of water. Nutrient composition of rat chow is reported at http://multipurina.ca/en/rodents/products/, nutrient composition of TetraColor granules is reported at http://www.oscarfish.com/1-star-foods/275-tetracolor-tropical-granules-ingredients-analysis.html, and nutrient composition of liver powder is reported at https://www.mpbio.com/includes/msds/02900396/MP_DS_02900396.pdf. The precise composition of dried yeast extract is undefined but predominantly consists of B vitamins amino acids, and proteins (ttps://www.sigmaaldrich.com/catalog/product/sial/70161?lang=en&region=US&cm_sp=Insite-_-prodRecCold_xviews-_-prodRecCold10-1). The rat chow and fish food diets were sterilized by gamma irradiation as previously described [5] while the liver power: yeast extract suspension was sterilized by autoclaving. A fourth diet combined 8 x 10^11^ *E*. *coli* in 5 ml of water with 1 ml of liver powder: yeast extract diet, 0.3 g of agar, and 19 ml of water, which was then autoclaved and poured into sterile plates to produce a solidified agar-based diet [28]. A fifth diet was identical to the fourth with the exception that it contained 8 x 10^11^ *E*. *coli* that were lysed by sonication before combining with the other components.

### Mosquitoes

The UGAL strain of *Ae*. *aegypti* was conventionally reared at 27° C, 70% relative humidity and a 16 h light-8 h dark photoperiod by feeding larvae rat chow diet and blood-feeding adult females on an anesthetized rat as described [31]. Sprague-Dawley strain rats from Charles Rivers Laboratories were anesthetized by trained personnel as in Animal Use Protocol A2018 12–013. The G3 strain of *Anopheles gambiae* was conventionally reared by feeding larvae the same diet or fish food while adult females were fed commercially purchased human blood (Valley Biomedical, Winchester, VA, USA) using an artificial feeder. Axenic larvae were produced from surface sterilized eggs [5]. Eggs were first placed in a sterile petri dish containing 70% ethanol in water for 5 min followed by transfer to a second petri dish containing 3% bleach and 0.1% ROCCAL-D (Pfizer) in sterile water for 3 min and a second wash in 70% ethanol for 5 min. Eggs were then placed in a new sterile petri dish and washed 3 times with 10 ml of sterile water followed by transfer to a 10 cm^2^ culture flask (Corning) containing 15 ml of sterile water for hatching. All assays using axenic larvae were thereafter conducted in a laminar hood with strict aseptic technique employed at all steps.

### Small scale rearing of mosquitoes and *E*. *coli* growth

Small scale rearing of *Ae*. *aegypti* was conducted in 6 well culture plates (Corning) containing 5 ml of sterile water per well. Approximately ten axenic larvae were added per well for a density of ~2 larvae per ml. Larvae in each well were inoculated with *E*. *coli* by adding 5 x 10^6^ bacterial cells (1 x 10^6^ per ml) to the water in each culture well (time 0) to produce gnotobiotic larvae. Larvae in other wells were inoculated by adding a drop of water from a regular culture of larvae (third instar) reared under non-sterile conditions to produce conventional larvae. Larvae were fed by adding sterile rat chow diet to the water in each well on the following schedule: 3.3 mg on days 1 and 2, and 8.3 mg of rat chow diet on days 4 and 5. Growth of *E*. *coli* in cultures containing *Ae*. *aegypti* larvae was monitored by sampling 10 μl of rearing water and determining the density of viable bacteria using the serial dilution method of Thomas et al. [32] on LB plates. Growth of *E*. *coli* in the absence of *Ae*. *aegypti* larvae was similarly assessed by inoculating wells with 1 x 10^2^ to 1 x 10^6^ *E*. *coli* per ml and following the rat chow diet and growth monitoring schedules described above.

### Larval molting, hypoxia responses, and nutrient acquisition

To assess the effects of bacterial density on growth of *Ae*. *aegypti* first instars, ~10 newly hatched axenic larvae were added to culture wells containing 5 ml sterile water. Larvae were then inoculated with *E*. *coli* by adding 1 x 10^2^–1 x 10^8^ living or heat killed cells to the water in each culture well. Larvae were then fed by adding 3.3 mg of rat chow diet to the water in each culture well. One cohort of larvae was monitored daily to determine the percentage of individuals that molted to the second instar. Other cohorts of larvae were collected 12 h post-inoculation in protease/phosphatase inhibitor cocktail and lysed as previously described [25]. Protein concentrations of resulting extracts were determined using Coomassie Plus Protein Reagent (ThermoFisher). Axenic larvae processed without exposure to food or bacteria served as the 0 h time point. Samples were resuspended in Laemmli buffer with mercaptoethanol (10 μM) followed by electrophoreses (30 μg per lane) on 4–20% Tris-Glycine gels (Biorad) and transfer to polyvinyl difluoride (PVDF) (ThermoFisher). After blocking in 5% non-fat dry milk in PBS+0.1%Tween 20 for 1 h, blots were probed with a rabbit anti-HIF-α antibody (1:5000) [25] or anti-actin (1:1000, A2103 Sigma Aldrich) that was used as a loading control. Samples were then washed and probed with a peroxidase-conjugated goat anti-rabbit secondary antibody (Jackson; 1:5000) followed by visualization using a chemiluminescent substrate (Clarity Western ECL Substrate, Biorad) and Syngene imaging system. Three immunoblots using independently acquired samples were run for each treatment. HIF-α abundance for each sample was estimated by densitometry using ImageJ software, which calculated in arbitrary units the density of the HIF-α and actin loading control bands to generate a HIF-α/actin ratio for each replicate.

Newly hatched axenic larvae (2–4 per ml) were placed into culture wells containing 5 ml of sterile water and inoculated by adding living or heat-killed *E*. *coli*, *S*. *cerevisiae*, *C*. *reinhardtii* (1 x 10^8^ per ml), or S2 cells (1 x 10^6^ per ml) to the water or medium with or without rat chow. One cohort of larvae was monitored daily until all larvae either molted to the second instar or died. Other cohorts were processed to measure gut hypoxia, HIF-α stabilization, and the number of neutral lipid droplets in the midgut and fat body at 24 h post-inoculation. Midgut hypoxia levels were quantified at 12 h post-inoculation using a Zeiss LSM 710 confocal microscope and Image iT Hypoxia reagent (Thermo Fisher) as previously described [21]. Stabilized HIF-α was visualized by immunoblotting as outlined above, while the number of neutral lipid droplets per midgut enterocyte or fat body adipocyte was measured at 15 h post-inoculation by Nile red staining (Molecular Probes) and counterstaining of cell nuclei with the DNA specific dye Hoechst 33342 (Sigma) [25].

### Larval growth when fed other diets

Newly hatched axenic larvae (2–4 per ml) were added to culture wells containing 5 ml of sterile water plus 23 mg of sterile fish food or 50 μl of liver powder: yeast extract diet. We then set up the same treatments but also inoculated larvae by adding to the water in culture wells either 1 x 10^6^ per ml of living *E*. *coli* or *S*. *cerevisiae*, 1 x 10^8^ or 10^9^ per ml whole heat-killed *E*. *coli* or *S*. *cerevisiase*, or 1 x 10^8^ or 10^9^ per ml *E*. *coli* or *S*. *cerevisiae* that were lysed by sonication before heat-killing. Culture wells with newly hatched axenic larvae were also fed 0.4 g of the liver powder: yeast extract: whole heat-killed *E*. *coli* agar diet or 0.4 g of the liver powder: yeast extract: lysed heat-killed *E*. *coli* agar diet. Each treatment was then monitored until all larvae either molted to the second instar or died. Cohorts of conventional larvae inoculated with a drop of water from a regular non-sterile culture were also fed rat chow diet, fish food or liver powder:yeast extract diet on a regular schedule were monitored until development into adults or death.

### Sterility assessment

For each assay, the presence of bacteria was monitored by plating aliquots of culture water on LB agar plates or using universal primers for the 16S rRNA gene by PCR as previously described [5, 18]. The presence of yeast and other fungi was monitored by plating aliquots of culture water on YPD or Potato Dextrose Agar (PDA) plates.

### Data analyses

Individual larvae were the unit of replication in assays that assessed the effects of different organisms or diets on the proportion of first instars that molted or developed into adults. For small scale rearing of axenic, gnotobiotic and conventional larvae the total number of larvae monitored for development into adults derived from hatching 10 cohorts of eggs which resulted in 10 biological replicates of ~60 larvae. The total number of larvae for each treatment that developed into adults was then summed across replicates to generate a total percentage of larvae per treatment that developed into adults. Resulting contingency data were then analyzed by Fisher Exact Tests. For the other assays in which the proportion of larvae that molted or developed into adults, each assay was conducted using larvae from one cohort of eggs that were hatched. Resulting contingency data were analyzed using Fisher Exact Tests. The effects of different treatments on hypoxia as measured by image iT fluorescence, HIF-α abundance as measured by densitometry, and the abundance of lipid droplets in midgut or fat body cells yielded continuous variables that were initially assessed for normality by Shapiro-Wilk W tests followed by one-way analysis of variance (ANOVA) and post-hoc Dunnett’s tests or Kruskal Wallis and post-hoc Dunn’s tests. All analyses were performed using the JMP Version 13 (SAS) statistical platform.

## Results

### *E*. *coli* rapidly grows under culture conditions used to rear mosquitoes and stabilizes HIF-α in inoculated larvae

**Fig 1:**
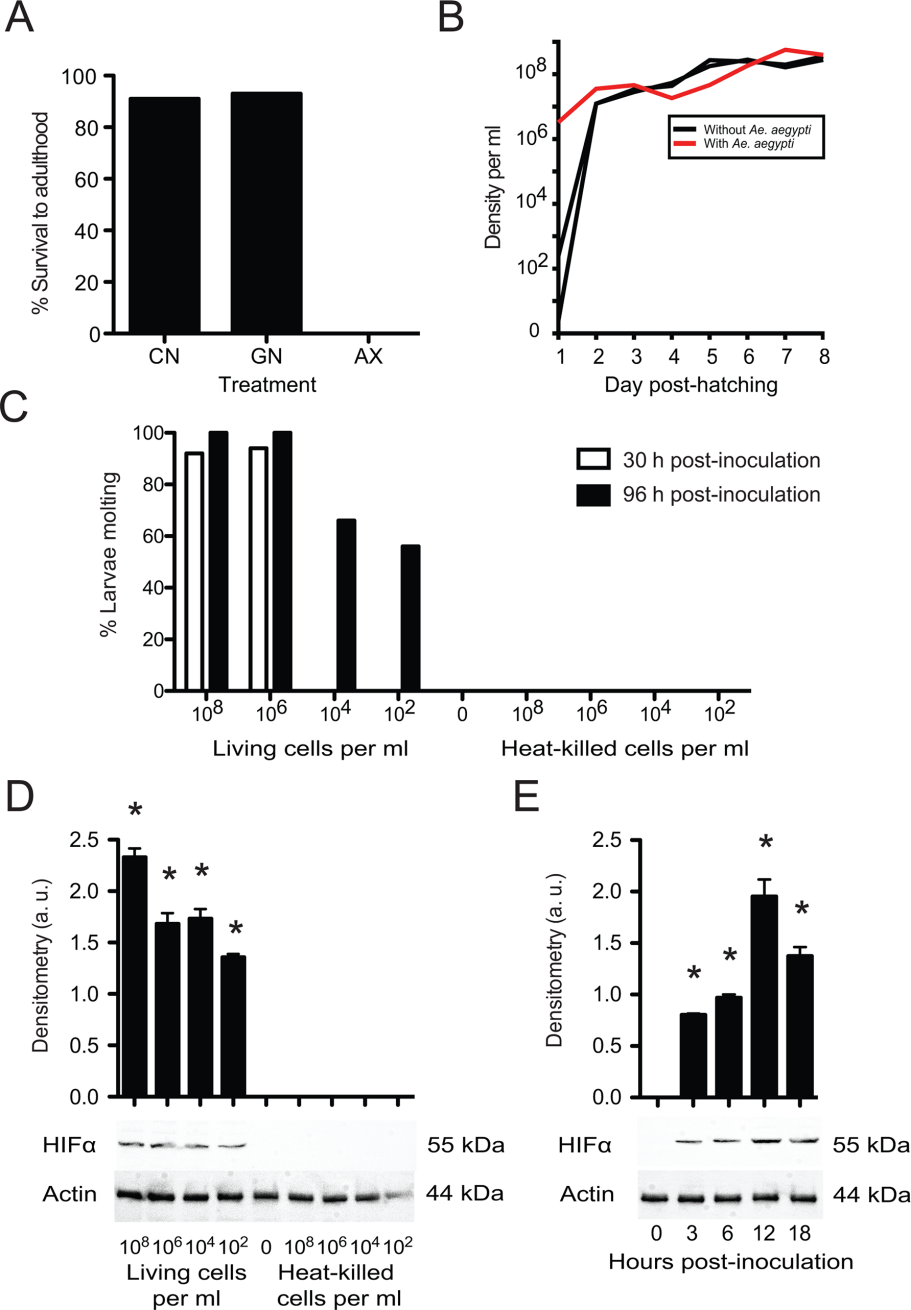
Growth of *Ae*. *aegypti* larvae fed rat chow diet and inoculated with no microbes, living microbes, or heat-killed microbes. (A) Percentage of axenic first instars (AX), gnotobiotic first instars (GN) inoculated with living *E*. *coli* (1 x 10^6^ per ml), or conventional first instars (CN) inoculated with water from a laboratory culture that developed into adults. A total of 600 first instars were monitored per treatment (10 culture plates, 10 larvae per well). A Fisher’s Exact Test detected strong differences among treatments (p<0.001) due to no AX larvae developing into adults. (B) Mean number of living *E*. *coli* per ml of water in culture wells inoculated with rat chow diet, 10 *Ae*. *aegypti* first instars, and 1 x 10^6^ bacteria per ml of water (red line), rat chow plus 10 bacteria per ml of water (black line), or rat chow plus 100 bacteria per ml of water (black line). (C) Percentage of axenic first instars (AX) that molted to the second instar by 30 or 96 h in cultures inoculated with rat chow plus different starting densities of living or heat-killed *E*. *coli*. A minimum of 60 first instars (10 larvae per culture well) per treatment were monitored until molting or death. A strong difference among treatments was detected (Fisher’s Exact Test; p<0.001) due to no larvae molting in cultures with no bacteria or inoculated with heat-killed *E*. *coli*. (D) Immunoblot from larvae fed rat chow diet and inoculated with different starting densities of living *E*. *coli*, heat-killed *E*. *coli* or no bacteria (control). Samples were collected 12 h post-inoculation and probed with anti-HIF-α and anti-actin which served as a loading control. Molecular masses of each protein are indicated to the right. The graph above the immunoblot shows mean density in arbitrary units (a. u.) of the HIF-α band relative to actin from three independently generated immunoblots. Each bar shows the mean value ± the standard deviation (SD). A Kruskal-Wallis test detected a strong difference among treatments (Χ^2^ = 25.6; P<0.0001) while asterisks indicate treatments that significantly differed (α = 0.05) from the no bacteria control (post-hoc Dunn’s test). (E) Immunoblot from larvae fed rat chow diet and inoculated with 1 x 10^8^ living *E*. *coli* per ml of water. Samples were collected 0–18 h post-inoculation and probed with anti-HIF-α or anti-actin as described above. The graph above the immunoblot shows mean density ± SD of the HIF-α band. Asterisks indicate treatments that significantly differed from the 0 h time point by ANOVA (F_4, 14_ = 216.2; P<0.0001) and a post-hoc Dunnett’s test (α = 0.05).

Under conventional rearing conditions used in our laboratory, UGAL strain *Ae*. *aegypti* are fed rat chow diet, which results in first instars molting to the second instar at ~24 h post-hatching, the third instar at ~48 h, and fourth instar at ~ 72 h. Larvae then pupate on day 5 or 6 and emerge as adults on day 7 or 8 [19,31]. In this study, we used 6 well culture plates for small-scale rearing of larvae and bioassays. As previously reported [5], no axenic larvae grew beyond the first instar in culture wells containing only sterile water and rat chow diet (Fig 1A). However, more than 90% of the larvae developed into adults by day 8 when inoculated with a drop of water from our main culture containing conventionally reared third instars, or 1 x 10^6^ living *E*. *coli* per ml of water to produce gnotobiotic larvae (Fig 1A). Since very similar percentages of conventional and *E*. *coli* gnotobiotic larvae developed into adults, we focused on the latter to assess the abundance of bacteria present in culture wells over the course of development. Results showed that *E*. *coli* increased from an inoculation density of 10^6^ living cells per ml to near 10^8^ living cells by day 2 with density thereafter remaining near constant until adult emergence (Fig 1B). *E*. *coli* also grew to similar densities when inoculated into cultures containing rat chow diet but no mosquito larvae, which indicated the carrying capacity for *E*. *coli* under these culture conditions is ~10^8^ per ml (Fig 1B).

We therefore next inoculated cultures with up to 10^8^ living or heat-killed *E*. *coli* cells per ml of water and assessed: 1) the proportion of axenic first instars that molted to the second instar, and 2) whether stabilized HIF-α was present since detection of this transcription factor subunit correlated with larvae that grew and molted in previous studies but was absent in larvae that did not [25]. Results showed that most larvae in cultures inoculated with 10^6^ or 10^8^ living *E*. *coli* per ml molted to the second instar within 30 h (Fig 1C). No larvae molted within 30 h in cultures inoculated with lower starting densities of living *E*. *coli* but many did so by 96 h (Fig 1C). Continued monitoring of these larvae also showed, as expected, that cultures inoculated with 10^6^ or 10^8^ living *E*. *coli* per ml resulted in most individuals emerging as adults on day 8 while larvae inoculated with lower starting densities of bacteria emerged after 12 or more days. In contrast, cultures inoculated with heat-killed *E*. *coli* over the same range of starting densities never molted to the second instar (Fig 1C). Immunoblot analysis detected HIF-α in larvae inoculated with living *E*. *coli* across all starting densities, whereas no HIF-α was detected in larvae inoculated with no *E*. *coli* or different starting densities of heat-killed *E*. *coli* (Fig 1D). At a starting density of 10^8^ living *E*. *coli* per ml, we first detected HIF-α at 3 h post-inoculation with abundance increasing at 12 h and then declining at 18 h which preceded molting to the second instar at ~24 h (Fig 1E). Culture assays confirmed that viable *E*. *coli* were present in all cultures inoculated with living bacteria, but no viable bacteria or fungi were detected in cultures without bacteria or inoculated with heat-killed *E*. *coli*. We thus concluded that living *E*. *coli* stimulated *Ae*. *aegypti* first instars to molt and stabilized HIF-α over a range of inoculation densities, whereas heat killed *E*. *coli* over the same range of starting densities did not.

### Other living organisms besides bacteria stimulate larval growth

To test whether other living organisms besides bacteria can induce *Ae*. *aegypti* larvae to grow, we conducted bioassays with: 1) *S*. *cerevisiae* and *C*. *reinhardtii* since related fungi and algae have been identified as gut community members in some mosquito species [4, 14–16], and 2) *Drosophila* S2 cells, given previous results showing that axenic larvae from two mosquito species grow when fed insect cells [27]. While *S*. *cerevisiae* and S2 cells are aerobic heterotrophs, *C*. *reinhardtii* is an aerobic autotroph [33].

**Fig 2:**
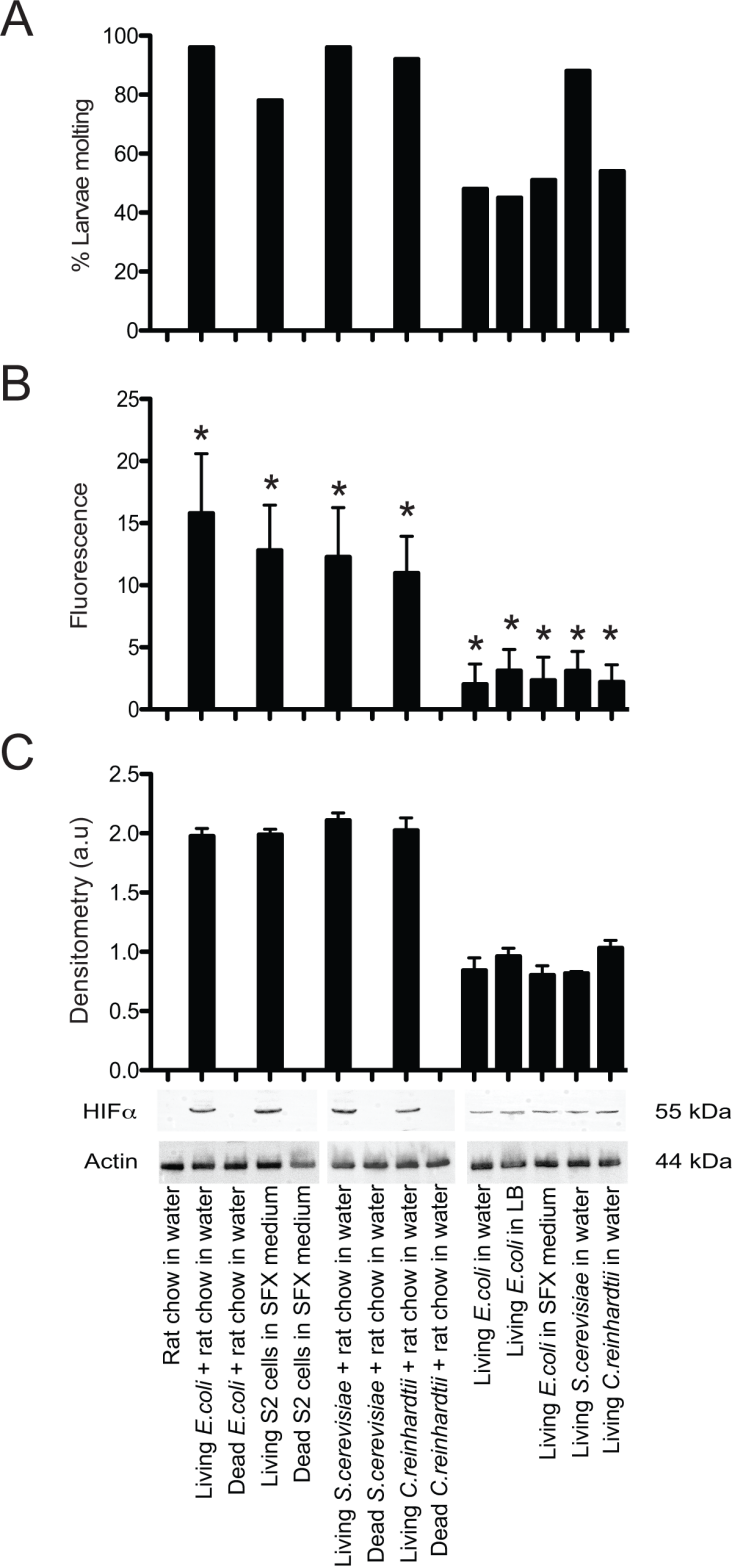
Growth of *Ae*. *aegypti* larvae inoculated with different living or heat-killed organisms. (A) Percentage of AX first instars that molted to the second instar when fed: rat chow diet alone, *E*. *coli*, *S*. *cerevisiae*, or *C*. *reinhardtii* plus rat chow diet, S2 cells in SFX medium, or *E*. *coli*, *S*. *cerevisiae*, or *C*. *reinhardtii* alone. A minimum of 60 first instars (10 larvae per culture well) were monitored daily per treatment until either molting to the second instar or death. A Fisher’s exact test identified significant treatment differences (p<0.001) due to no larvae molting in cultures fed rat chow diet alone or rat chow diet plus heat-killed organisms. (B) Quantification of Image iT fluorescence in the midguts of larvae. Twenty larvae per treatment were examined by confocal microscopy at 12 h post-inoculation. Bars in the graph show mean pixel intensity values ± SD. A Kruskal-Wallis detected a strong difference among treatments (Χ^2^ = 260.9; P<0.0001) while asterisks indicate treatments that significantly differed (α = 0.05) from larvae fed only rat chow diet (post-hoc Dunn’s test). (C) Immunoblots of first-instar extracts prepared from larvae collected 12 h post-inoculation. Samples were probed with anti-HIF-α or anti-actin with the graph above the immunoblots showing mean density ± SD in arbitrary units (a. u.) of the HIF-α band relative to the actin loading control from three independently generated immunoblots.

Earlier experiments showed that cultures inoculated with different species of living bacteria but no food results in some *Ae*. *aegypti* first instars molting to the second instar [5]. However, these larvae soon after died, which resulted in no larvae inoculated with bacteria developing into adults if no other food source like rat chow diet was provided. We therefore first compared the proportion of axenic first instars in water that molted to the second instar with or without rat chow diet when inoculated with 10^8^ per ml of living or heat-killed *E*. *coli*, *S*. *cerevisiae* or *C*. *reinhardtii*. We also assessed the proportion of larvae that molted when provided 10^6^ per ml of living or heat killed S2 cells in SFX medium or living *E*. *coli* (10^6^ per ml) in SFX or LB medium. Axenic larvae in water containing rat chow diet only served as a negative control. Results showed that almost all first instars inoculated with living *S*. *cerevisiae*, *C*. *reinhardtii*, or *E*. *coli* and fed rat chow diet molted to the second instar (Fig 2A). Most larvae fed living S2 cells molted while ~50–80% of first instars inoculated with living *S*. *cerevisiae*, *C*. *reinhardtii*, or *E*. *coli* alone also molted (Fig 2A). In contrast, no larvae molted in cultures that contained rat chow diet only, rat chow diet plus heat-killed microbes, or heat-killed S2 cells in SFX medium (Fig 2A). We next measured gut hypoxia using Image iT, which fluoresces with increasing intensity as atmospheric oxygen concentration falls below 5% [18], and stabilized HIF-α at 12 h post-inoculation across all treatments. iT fluorescence and HIF-α abundance values were highest in larvae inoculated with living *E*. *coli*, *S*. *cerevisiae*, or *C*. *reinhardtii*, plus rat chow diet, and living S2 cells in SFX medium (Fig 2B and 2C). Image iT fluorescence and HIF-α abundance were lower but detectable in larvae inoculated with living *S*. *cerevisiae*, *C*. *reinhardtii*, or *E*. *coli* but no rat chow diet, while no iT fluorescence or HIF-α were detected in larvae inoculated with heat-killed microbes plus rat chow diet or heat killed S2 cells in SFX medium (Fig 2B and 2C). Culture-based assays on LB or YPD plates indicated the presence of living bacteria or fungi in all treatments inoculated with living *E*. *coli* or *S*. *cerevisiae*, and the absence of living bacteria or yeast in all treatments that were inoculated with dead or no organisms.

**Fig 3:**
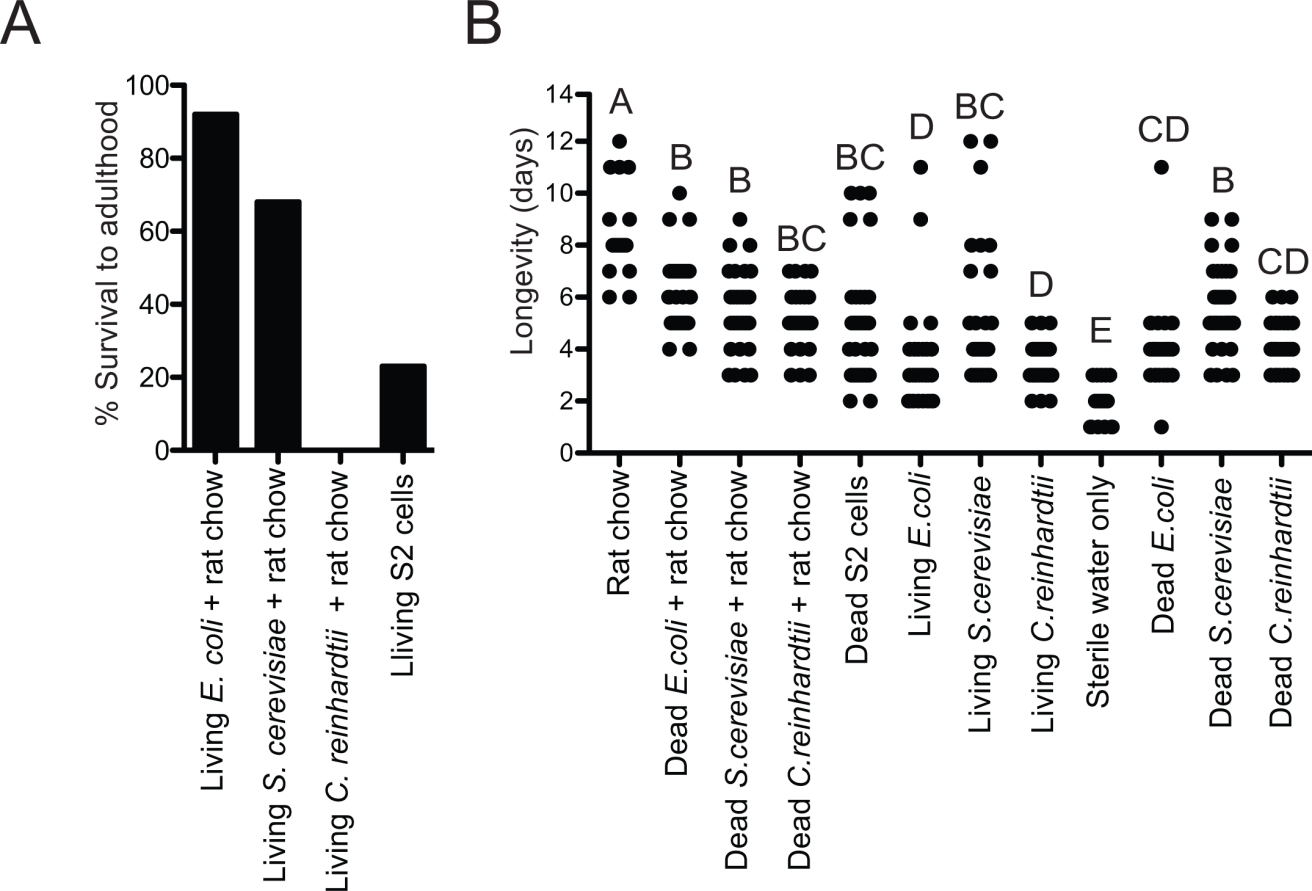
Development into adults or longevity of *Ae*. *aegypti* larvae inoculated with different living or heat-killed organisms. (A) Percentage of AX first instars that developed into adults when fed rat chow diet and inoculated with living *E*. *coli*, *S*. *cerevisiae*, *C*. *reinhardtii*, or fed S2 cells in SFX medium. A minimum of 60 first instars were monitored per treatment. A Fisher’s exact test identified significant treatment differences (p<0.001) due to few or no larvae inoculated with *C*. *reinhardtii* or fed S2 cells developing into adults. (B) Longevity of axenic first instars fed rat chow diet in water, rat chow diet in water plus heat-killed *E*. *coli*, *S*. *cerevisiae*, *C*. *reinhardti*, heat-killed S2 cells in SFX medium, or living or heat-killed *E*. *coli*, *S*. *cerevisiae* or *C*. *reinhardti* in water. The longevity of axenic larvae in sterile water with no food or microbes was also examined. A minimum of 30 larvae was monitored per treatment. ANOVA and a post-hoc Tukey Kramer HSD test (α = 0.05) compared longevity among treatments. Different letters indicate treatments that significantly differed from one another (F_10, 374_ = 68.1; P<0.0001).

Given the preceding results, we monitored larvae across treatments until death or growth to the adult stage. Most larvae inoculated with living *E*. *coli* or *S*. *cerevisiae* and fed a regular schedule of rat chow diet developed into adults (Fig 3A). No larvae inoculated with living *C*. *reinhardtii* and fed rat chow diet developed into adults (Fig 3A) although most molted to the third or fourth instar before dying. Some larvae fed S2 cells in SFX medium daily developed into adults but most died as third or fourth instars (Fig 3A). While no larvae fed rat chow diet only, rat chow diet plus dead microbes, or dead S2 cells molted (see above), most first instars in these treatments remained alive for more than 4 days and some lived up to 12 days (Fig 3B). This was in marked contrast to axenic first instars in water only, which all died within 3 days (Fig 3B). First instars inoculated with living *S*. *cerevisiae*, *C*. *reinhardtii*, or *E*. *coli* daily but no rat chow diet lived longer than axenic larvae in water only but not as long as axenic larvae fed rat chow diet (Fig 3B). Most larvae inoculated with living microbes but with no rat chow diet never molted beyond the second instar although the three longest living individuals inoculated with living *S*. *cerevisiae* molted to the third instar before dying (Fig 3B). We also monitored the longevity of axenic first instars inoculated with dead microbes only at a density of 10^8^ per ml. No larvae in these treatments ever molted but most lived longer than axenic larvae in water only (Fig 3B).

### Larvae acquire neutral lipids when inoculated with different living organisms

**Fig 4:**
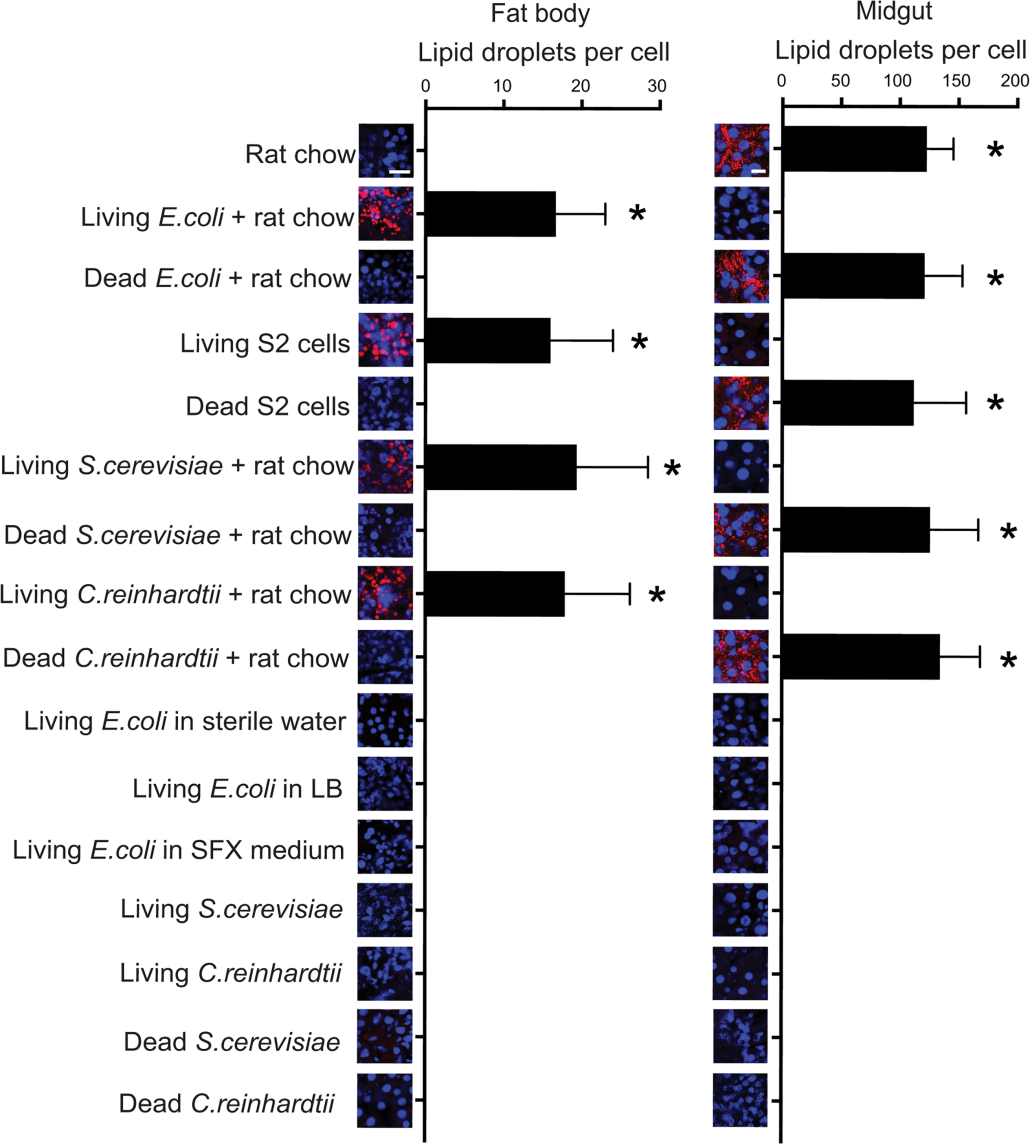
Living organisms and food affect neutral lipid droplet accumulation in the midgut and fat body of *Ae*. *aegypti* larvae. Confocal images of fat body adipocytes (left panel) and midgut enterocytes (right panel) from larvae fed rat chow diet in water (negative control), living or heat-killed *E*. *coli*, *S*. *cerevisiae*, or *C*. *reinhardtii* in water plus rat chow, living or heat-killed S2 cells in SFX medium, living *E*. *coli* in water, LB or SFX medium, and living or heat-killed *S*. *cerevisiae* or *C*. *reinhardtii* in water. All larvae were collected at 15 h post-inoculation. For each image, lipid droplets stained with Nile red are red while cell nuclei stained with Hoechst 33342 are blue. Scale bars for the midgut and fat body images equal 20 μm. The graphs in each panel show the mean number (± SD) of neutral lipid droplets per midgut enterocyte or fat body adipocyte. Kruskal-Wallis tests indicated that droplet abundance in enterocytes (Χ^2^ = 154.9; P<0.0001) or adipocytes (Χ^2^ = 152.1; P<0.0001) significantly differed among treatments. Asterisks above a given treatment indicate that the number of neutral lipid droplets present significantly differed (α = 0.05) from larvae fed rat chow diet alone.

Studies of several insects indicate that neutral lipids biosynthesized in the midgut from consumed nutrients are transported by the carrier protein lipophorin to the fat body where they serve as a primary source of energy for growth [34–36]. We previously reported that the fat body in conventionally reared first instars and gnotobiotic first instars inoculated with living *E*. *coli* accumulate neutral lipid droplets when fed rat chow diet [25]. In contrast, axenic larvae fed rat chow diet alone accumulate neutral lipids in the midgut but show no accumulation in the fat body. These observations together with functional experiments supported a role for hypoxia-induced HIF signaling in stimulating neutral lipid transport from midgut enterocytes to fat body adipocytes [25]. Larvae fed rat chow diet and inoculated with living *E*. *coli*, *S*. *cerevisiae* or *C*. *reinhardtii*, as well as larvae fed S2 cells in SFX medium contained very similar numbers of neutral lipid droplets in fat body adipocytes at 15 h post-inoculation (Fig 4). In contrast, larvae fed rat chow diet and inoculated with dead *E*. *coli*, *S*. *cerevisiae*, or *C*. *reinhardtii*, or dead S2 cells in SFX medium showed no accumulation of neutral lipid droplets in fat body adipocytes but an abundance of neutral lipid droplets in midgut enterocytes (Fig 4). Strikingly, larvae inoculated with living *E*. *coli*, *S*. *cerevisiae*, or *C*. *reinhardtii* but with no rat chow diet showed no neutral lipid accumulation in either the midgut or fat body at 15 h post-inoculation (Fig 4).

### *Ae*. *aegypti* fed other diets require living organisms to grow

Since axenic *Ae*. *aegypti* fed rat chow diet only grew if living organisms were present, we assessed whether feeding two other diets (fish food and liver powder: yeast extract) altered this requirement. Results showed that no axenic first instars ever molted when fed each diet alone, each diet plus 10^8^ or 10^9^ per ml of heat-killed *E*. *coli* or *S*. *cerevisiae*, or each diet plus 10^8^ or 10^9^ per ml of *E*. *coli* or *S*. *cerevisiae* that were lysed before heat-killing (Table 1). In addition, no axenic larvae molted when fed liver powder: yeast extract plus dead or lysed *E*. *coli* embedded in agar (Table 1). In contrast, nearly all larvae molted to the second instar within 30 h when inoculated with 10^6^ per ml living *E*. *coli* or living *S*. *cerevisiae* and fed the same diets (Table 1). Similar to axenic *Ae*. *aegypti* first instars fed rat chow diet, more than 30% of axenic first instars fed fish food or liver power: yeast extract diet lived more than 8 days even though they never molted. We therefore asked whether first instars after 8 days in cultures with rat chow diet, fish food, or liver power: yeast extract diet alone molt when inoculated with living microbes. Results showed that adding living *E*. *coli*, *S*. *cerevisiae*, or a mixed community of microbes from our main laboratory culture induced most larvae to molt to the second instar within 36 h of inoculation (Table 2). Continued feeding of larvae after inoculation with a conventional community of microbes further resulted in most individuals developing into adults (Table 3).

**Table 1:** Percentage of newly hatched *Ae*. *aegypti* first instars that molted to the second instar when fed fish food or a liver powder: Yeast extract diet alone or supplemented with living, dead, or lysed microbes.

Treatment	N	% Larvae molting to the second instar
**Fish food**	**60**	**0**
**Liver powder: yeast extract**	**60**	**0**
**Fish food + 10**^**8**^ **dead *E*. *coli***	**62**	**0**
**Fish food + 10**^**9**^ **dead *E*. *coli***	**60**	**0**
**Fish food + 10**^**8**^ **lysed *E*. *coli***	**63**	**0**
**Fish food + 10**^**9**^ **lysed *E*. *coli***	**61**	**0**
**Fish food + 10**^**6**^ **living *E*. *coli***	**64**	**100**
**Fish food + 10**^**8**^ **dead *S*. *cerevisiae***	**60**	**0**
**Fish food + 10**^**9**^ **dead *S*. *cerevisiae***	**60**	**0**
**Fish food + 10**^**8**^ **lysed *S*. *cerevisiae***	**60**	**0**
**Fish food + 10**^**9**^ **lysed *S*. *cerevisiae***	**60**	**0**
**Fish food + 10**^**6**^ **living *S*. *cerevisiae***	**60**	**98**
**Liver powder: yeast extract + 10**^**8**^ **dead *E*. *coli***	**60**	**0**
**Liver powder: yeast extract + 10**^**9**^ **dead *E*. *coli***	**60**	**0**
**Liver powder: yeast extract + 10**^**8**^ **lysed *E*. *coli***	**60**	**0**
**Liver powder: yeast extract + 10**^**9**^ **lysed *E*. *coli***	**60**	**0**
**Liver powder: yeast extract + 10**^**6**^ **living *E*. *coli***	**62**	**100**
**Liver powder: yeast extract + 10**^**8**^ **dead *S*. *cerevisiae***	**61**	**0**
**Liver powder: yeast extract + 10**^**9**^ **dead *S*. *cerevisiae***	**60**	**0**
**Liver powder: yeast extract + 10**^**8**^ **lysed *S*. *cerevisiae***	**62**	**0**
**Liver powder: yeast extract + 10**^**9**^ **lysed *S*. *cerevisiae***	**62**	**0**
**Liver powder: yeast extract + 10**^**6**^ **living *S*. *cerevisiae***	**60**	**100**
**Agar embedded liver powder: yeast extract + dead *E*. *coli***	**60**	**0**
**Agar embedded liver powder: yeast extract + lysed *E*. *coli***	**60**	**0**

N equals the number of first instars per treatment that were monitored daily until molting to the second instar or death.

**Table 2:** Percentage of 8 day old axenic *Ae*. *aegypti* first instars fed different diets that molted to the second instar after inoculation with living microbes.

Treatment	N	% Larvae molting to the second instar
**Rat chow (negative control)**	**60**	**0**
**Fish food (negative control)**	**64**	**0**
**Liver powder: yeast extract (negative control)**	**61**	**0**
**Rat chow + 10**^**6**^ **living *E*. *coli***	**61**	**97**
**Rat chow + 10**^**6**^ **living *S*. *cerevisiae***	**63**	**97**
**Rat chow + conventional culture**	**61**	**98**
**Fish food + 10**^**6**^ **living *E*. *coli***	**64**	**97**
**Fish food + 10**^**6**^ **living *S*. *cerevisiae***	**60**	**93**
**Fish food + conventional culture**	**60**	**98**
**Liver powder: yeast extract + 10**^**6**^ **living *E*. *coli***	**60**	**97**
**Liver powder: yeast extract + 10**^**6**^ **living *S*. *cerevisiae***	**60**	**95**
**Liver powder: yeast extract + conventional culture**	**60**	**100**

N equals the number of first instars per treatment that were monitored daily until molting to the second instar or death.

**Table 3:** Percentage of 8 day old axenic *Ae*. *aegypti* first instars fed different diets that developed into adults after inoculation with living microbes from a conventional culture.

Treatment	N	% Larvae molting to the second instar
**Rat chow + conventional culture**	**61**	**88**
**Fish food + conventional culture**	**60**	**82**
**Liver powder: yeast extract + conventional culture**	**60**	**86**

N equals the number of first instars per treatment that were monitored daily until emerging as an adult or death.

### *An*. *gambiae* also requires living microbes to grow

Lastly, we conducted bioassays with axenic *An*. *gambiae* larvae, which we previously reported to also not grow beyond the first instar if fed only sterilized rat chow diet [5]. Elaborating on these findings, no axenic *An*. *gambiae* first instars ever molted if fed rat chow diet supplemented with 10^8^ or 10^9^ per ml heat-killed *E*. *coli* or *S*. *cerevisiae*, or 10^8^ or 10^9^ per ml *E*. *coli* or *S*. *cerevisiae* that were lysed by sonication before autoclaving (Table 4). In contrast, most axenic *An*. *gambiae* molted within 36 h if fed rat chow diet and inoculated with 10^8^ per ml living *E*. *coli* or 10^6^ per ml living *S*. *cerevisiae* (Table 4). Across all of the assays we conducted using different diets with *Ae*. *aegypti* or *An*. *gambiae*, plate assays confirmed that no living bacteria or fungi were present in treatments that lacked living microbes while living bacteria and yeast were present in cultures that we inoculated with living *E*. *coli* or *S*. *cerevisiae*.

**Table 4:** Percentage of newly hatched *An*. *gambiae* first instars that molted to the second instar when fed different diets alone or supplemented with living or dead microbes.

Treatment	N	% Larvae molting to the second instar
**Rat chow**	**60**	**0**
**Rat chow + 10**^**8**^ **dead *E*. *coli***	**60**	**0**
**Rat chow + 10**^**9**^ **dead *E*. *coli***	**61**	**0**
**Rat chow + 10**^**8**^ **lysed *E*. *coli***	**64**	**0**
**Rat chow + 10**^**9**^ **lysed *E*. *coli***	**60**	**0**
**Rat chow + 10**^**6**^ **living *E*. *coli***	**62**	**87**
**Rat chow + 10**^**8**^ **dead *S*. *cerevisiae***	**60**	**0**
**Rat chow + 10**^**9**^ **dead *S*. *cerevisiae***	**64**	**0**
**Rat chow + 10**^**8**^ **lysed *S*. *cerevisiae***	**60**	**0**
**Rat chow + 10**^**9**^ **lysed *S*. *cerevisiae***	**60**	**0**
**Fish food + 10**^**6**^ **living *S*. *cerevisiae***	**65**	**92**

N equals the number of first instars per treatment that were monitored daily until molting to the second instar or death.

## Discussion

Our previous studies using bacteria and rat chow diet indicated that larvae require living microbes for growth [5]. Prior results further showed that: 1) larvae inoculated with wild-type *E*. *coli* or other bacteria exhibit midgut oxygen levels below 5%, 2) larvae exhibiting gut hypoxia express stabilized HIF-α, and 3) HIF agonists activate several processes with growth functions while HIF antagonists inhibit them [21,25]. Here, we conducted studies that assessed whether previous results are affected by bacterial density, whether ingestion of other aerobic organisms besides bacteria can induce larvae to grow, and if certain diets eliminate a requirement for living organisms in the gut for growth.

The first part of our study focused on culture conditions used in previous studies to show that *E*. *coli* grow to a similar density (~10^8^ living cells per ml) in the presence and absence of *Ae*. *aegypti* larvae (Fig 1). As expected, inoculation density affected how long it takes *E*. *coli* to reach this titer, which in turn affects the duration of the first instar, HIF-α abundance detected at 12 h, and development time into adults. However, heat-killed bacteria have no effect on larval growth over the same range of starting densities (Fig 1). These results fully support prior findings but add new information by showing that *E*. *coli* titers grow to ~10^8^ cells per ml under conditions used in earlier studies [5,18,25] and demonstrating that larval growth rates are only affected by the density of living bacteria that are present in *Ae*. *aegypti* cultures fed rat chow diet.

We previously proposed that other aerobically respiring organisms in the mosquito gut besides bacteria could induce larvae to grow by activating a gut hypoxia signal [21]. The second part of our study provides strong support for this by showing that a living yeast, alga, and an insect cell line each induce axenic *Ae*. *aegypti* first instars to molt, stimulated similar levels of midgut hypoxia, induced HIF-α stabilization, and promoted neutral lipid accumulation in the fat body (Figs 2–4). In contrast, no axenic larvae molted, exhibited a hypoxia response, or accumulated neutral lipids in the fat body when inoculated with the same organisms if heat-killed. The third part of our study further shows that no axenic larvae molted when fed two other diets, diets supplemented with heat-killed microbes or diets supplemented with lysed and heat-killed microbes at a titer equal to (10^8^ cells per ml) or greater (up to 10^11^ cells per ml) than the titer *E*. *coli* grows to in cultures fed rat chow diet (Table 1). Assays with *An*. *gambiae* further yielded similar outcomes (Table 4).

The common features shared by the microbes and S2 cells we bioassayed are each induced a gut hypoxia response and larval growth if viable. Our results further suggest none of the diets we tested are nutritionally deficient or that adding heat-killed microbes provides missing factors since none supported growth in the absence of living organisms but each did so when living organisms were present. These conclusions are broadly consistent with a recent study that found no differences in growth and adult fitness of conventionally reared *Ae*. *aegypti* from cultures fed a rat chow-based diet versus a liver powder-based diet recommended by the International Atomic Energy Agency [37]. However, our results greatly differ from Correa et al. [28] who report rearing axenic *Ae*. *aegypti* to the adult stage on diets that yielded no growth beyond the first instar in our experiments. Such opposite outcomes suggest either *Ae*. *aegypti* larvae substantially vary between laboratory cultures or populations in their requirements for growth or differences in methodology. Previous findings [5,18] as well as results from the current study do not indicate that different populations of *Ae*. *aegypti* or mosquito species differ in their requirement for living microbes to grow. We thus conclude methodology more likely underlies the differences between our findings and those of Correa et al. [28].

Our results from the current study indicate that the density of microbes in a culture affect larval growth rates and that several organisms besides bacteria can induce axenic larvae to grow even after many days of persisting in cultures with no microbes. Thus, risks of contamination by different types of microbes at either the onset of establishing a culture or later can both result in larvae growing. We have on several occasions observed what we thought were axenic larvae growing that we did not inoculate with a known organism, but in every case downstream studies identified contaminants that most commonly were fungi but also other microbes including bacteria. Through previous studies we also know that a number of bacteria in our conventional mosquito cultures as well as in mosquitoes collected from the field exhibit high levels of resistance to different classes of antibiotics [18]. Thus, antibiotic treatment of field collected or conventionally reared larvae often does not eliminate all bacteria or antibiotic insensitive unicellular eukaryotes. Our previously reported approach for producing axenic first instars by surface sterilizing eggs in contrast very reliably produces axenic larvae [5]. However, contamination can still sometimes occur through low level survival of bacteria or fungal spores. Reducing the number of eggs that are surface sterilized per batch and minimizing the volume of water axenic larvae are transferred in can both help further reduce the risk of contaminating axenic cultures. Altogether, our current findings indicate that strategies to produce axenic adult mosquitoes will either require rearing larvae by feeding them cell lines as originally proposed by Munderloh et al. [27] or using unicellular microbes during the larval stage that can be fully eliminated before pupation or after adult emergence.

Previous results indicated that bacteria-induced gut hypoxia and HIF signaling activate the insulin-insulin growth factor signaling (IIS) pathway and lipophorin expression which are both important in transport of neutral lipids by the fat body [25,34,35,38–40]. That down-regulated HIF signaling results in lipid accumulation in the midgut but no transport to the fat body previously suggested a role for bacteria in either directly modulating lipid biosynthesis by the midgut or indirectly modulating lipid biosynthesis through effects on other processes [25]. Results from the current study broaden this view by showing that larvae inoculated with living *E*. *coli*, *S*. *cerevisiae* or *C*. *reinhardtii* plus rat chow diet very similarly accumulated neutral lipids in the fat body, larvae fed rat chow diet with or without dead microbes only accumulate neutral lipids in the midgut, and larvae inoculated with living microbes alone accumulated no neutral lipids within 15 h of inoculation (Fig 4). These outcomes strongly suggest that neutral lipid loading in the gut does not depend on living bacteria specifically but does suggest that hypoxia and HIF signaling play important regulatory roles in processes required for normal neutral lipid transport. They further suggest that neutral lipids which accumulate in the midgut derive primarily from the diet larvae were fed rather than the microbe they were inoculated with. This could reflect the inability of larvae to efficiently digest these organisms, which all possess cell walls comprised of complex carbohydrates. However, it could also be affected by lower levels of nutrient absorption given evidence that larval mosquito midgut function is impaired in the absence of solid ingesta, which the diets we used provide [41]. Midgut dysfunction or lower level metabolism by microbes in cultures without food could also underlie why we detected lower levels of gut hypoxia and HIF-α in larvae inoculated with living microbes without food versus cultures containing living microbes plus food. In contrast, larvae accumulated neutral lipids in the fat body when fed living S2 cells with no rat chow diet which indicates these cells lived long enough after ingestion to induce a midgut hypoxia response but also provide nutrients for neutral lipid biosynthesis through the ability of larvae to digest them.

A final important observation from the current study is that axenic larvae in water never lived more than 3 days while larvae fed rat chow diet or certain heat-killed organisms in the absence of another food source often lived more than 8 days even though they never molted (Fig 3A). Thus, while larvae fed rat chow diet show defects in neutral lipid accumulation by the fat body, their midguts must acquire nutrients in the absence of a gut microbiota that extend longevity. Reciprocally, larvae fed living microbes but no food sometimes molted but usually died sooner than larvae fed only rat chow diet, while larvae fed rat chow diet plus *C*. *reinhardtii* or S2 cells almost always molted but rarely developed into adults. Thus, all of the organisms we bioassayed induced a gut hypoxia response and stimulated larvae to grow and molt to at least the second instar, but also exhibited differences in supporting growth into adults. This outcome could reflect variation in long-term viability of the organisms under the culture conditions we used in this study or potential differences in the types and/or availability of nutrients they provide in conjunction with the diets we used. Other studies of mosquitoes also identify differences in pupation rate and adult fitness traits in association with inoculation of gnotobiotic mosquitoes with different organisms [20,42]. Unlike mosquitoes, gut microbes are not essential for survival of terrestrial organisms like *Drosophila melanogaster* and mice because axenic cultures can be maintained over multiple generations if fed a nutritionally complete diet [43,44]. Yet similar to mosquito larvae, experimental studies indicate the absence of gut microbes is lethal for aquatic species from diverse taxa including *Hydra*, *Daphnia*, and zebrafish [45–48]. This suggests the possibility that the constant exposure to microbes in aqueous environments has potentially selected for greater dependence on a gut microbiota for growth. Greater understanding of the gut function with and without living microbes in mosquitoes and other aquatic organisms, and the factors underlying why certain microbes support development better than others are important questions for future study.
